# Cholesterol business: life or death by rust

**DOI:** 10.1038/s41392-024-01802-7

**Published:** 2024-03-21

**Authors:** Shubhangi Gavali, Francesca Maremonti, Andreas Linkermann

**Affiliations:** 1 Division of Nephrology, Department of Internal Medicine 3, University Hospital Carl Gustav Carus at the Technische Universität Dresden, Dresden, Germany; 2 Division of Nephrology, Department of Medicine, Albert Einstein College of Medicine, Bronx, NY USA

**Keywords:** Cell biology, Kidney diseases

In two recent manuscripts published in *Nature*, the cholesterol-synthesis pathway was identified as a master regulator of ferroptosis sensitivity and therefore as a novel therapeutic target.^1,2^ Ferroptosis does not only represent one way to eliminate cancer cells, but also contributes to acute organ damage in the kidney, heart, brain, and liver, and it kills tissues during the process of organ transplantation.

The term “ferroptosis” should be defined as a cellular process of iron-catalyzed lipid peroxidation-mediated rupture of the plasma membrane.^3^ In contrast to well-defined signaling pathways of regulated necrosis, such as necroptosis and pyroptosis, ferroptosis does not follow a pathway dominated by protein-to-protein communicated signal transduction, such as phosphorylation during necroptosis or protein cleavage during pyroptosis.^4^ For this simple but important reason, still no adequate biomarker allows for the detection of ferroptosis in tissue samples. However, the experimental evidence for ferroptosis as a driver of pathology in multiple diseases is overwhelming,^3^ and therefore, the unmet medical need to prevent ferroptosis is likely to outclass the other pathways.

Freitas et al.^1^ and Li et al.^2^ now independently identified 7-dehydrocholesterol reductase (DHCR7) as a key regulator of ferroptosis sensitivity. DHCR7 is involved in the final step of cholesterol biosynthesis where it reduces the double bond in the sterol B-ring of 7-dehydrocholesterol (7-DHC) to produce cholesterol. To identify genes that may potentially influence ferroptosis sensitivity, both the research groups performed genome-wide CRISPR-Cas9 screening in cells treated with the GPX4 inhibitor RSL3 and found *DHCR7* as one of the top-scoring hits. In vitro experiments using *DHCR7*^*-/-*^ cells exhibited elevated levels of 7-DHC and significant protection against RSL3-induced ferroptosis. This protective effect was abolished by deleting sterol C5-desaturase (SC5D), an enzyme crucial for 7-DHC synthesis. Importantly, the deletion of DHCR7 or SC5D had no impact on protein expression levels of known ferroptosis regulators such as ACSL4, GPX4, or FSP1. Additionally, the levels of glutathione and Fe^2+^ remained unaffected upon DHCR7 deletion. B-ring 5,7-diene is a characteristic feature of 7-DHC. To test the contribution of this unique molecular structure in preventing phospholipid autoxidation, both groups performed FENIX assays that allow to directly detect the potency of a potential radical trapping antioxidant (RTA). Preloading of liposomes with 7-DHC and sterols containing the B-ring 5,7-diene, such as ergosterol and 7-dehydrodemosterol, significantly blocked the phospholipid peroxidation, whereas cholesterol failed to do so. This result strongly suggested the important function of the B-ring 5,7-diene of 7-DHC as a hydrogen atom donor, which shields the phospholipids from peroxidation and consequently suppresses ferroptosis. Li et al., through the use of the cholesterol-synthesis pathway inhibitor Tasin-30, and Freitas et al., through the use of *DHCR7*^*-/-*^ mouse models and Tasin-1, addressed the 7-DHC-mediated anti-ferroptotic activity in certain tumors. Both these approaches demonstrated that reducing 7-DHC levels inhibited the growth of cancer cells, particularly those expressing high concentrations of 7-DHC. To further investigate the abundance of the 7-DHC anti-ferroptotic effect, Li et al. used the DHCR7 inhibitor AY9944. Treatment with AY9944 led to the accumulation of 7-DHC and resistance to ferroptosis in wild-type cells, but not in cells deficient in *SC5D*^*-/-*^. Given the importance of ferroptosis in renal ischemia-reperfusion injury (IRI), Li et al. also investigated AY9944 in the model of clamp ischemia of the kidneys. Remarkably, this tool compound significantly reduced structural organ damage of the renal tubular compartment and prevented the increase of serum urea and serum creatinine usually characteristic of this model. However, the *DHCR7*^*-/-*^ mice introduced in the Freitas et al. manuscript have not been used to confirm the relevance of this mechanism in renal IRI or the specificity of AY9944. However, collectively, these data indicate a potent anti-ferroptotic function of 7-DHC.

**Fig. 1:**
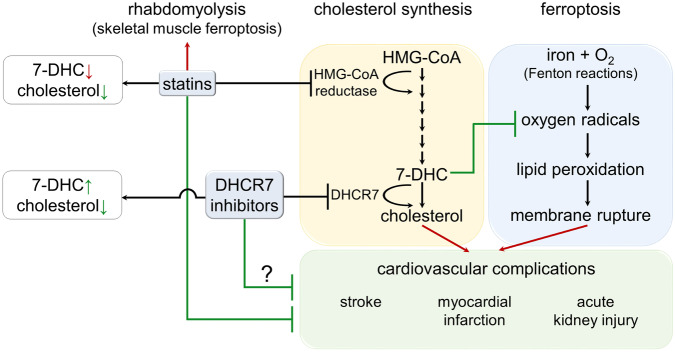
7-Dehydrocholesterol (7-DHC) functions as an endogenous regulator of ferroptosis sensitivity. The final step in the cholesterol-synthesis pathway is catalyzed by 7-dehydrocholesterol reductase (DHCR7) to generate cholesterol from 7-DHC. Identified through a CRISPR-Cas9 knockout screening approach, DHCR7 deficiency was identified to exert resistance to plasma membrane rupture by ferroptosis. Inhibition of DHCR7, therefore represents a novel therapeutic target for ferroptosis-mediated diseases, but the consequences of cholesterol-synthesis inhibition need to be carefully considered. Finally, DHCR7-inhibitors may be considered as cholesterol-lowering drugs, such as HMG-CoA reductase inhibitors, but additionally encompass an intrinsic anti-ferroptotic activity

A potentially significant comment may expand on the importance of these findings. HMG-CoA reductase inhibitors (statins) are broadly used to treat or prevent cardiovascular diseases. The most common typical side effect of statins is rhabdomyolysis. Ferroptosis was ascribed a causative role in the pathophysiology of rhabdomyolysis^5^ and it will be interesting to investigate 7-DHC levels in muscle biopsies of statin-treated patients that suffer from this particular condition which regularly leads to discontinuation of the otherwise beneficial treatment. This observation, together with results published in the two manuscripts, indicate that the benefit of statins may unfold despite the potentially 7-DHC-lowering effect of HMG-CoA reductase inhibitors. As demonstrated in Fig. 1, the direct inhibition of DHCR7 theoretically could copy the beneficial cholesterol-lowering effect of statins, but in addition might result in higher concentrations of 7-DHC and therefore in an intrinsic anti-ferroptotic function. This might not only overcome the potential ferroptosis-driven side effect of rhabdomyolysis, but also provide protection upon cardiovascular complications such as myocardial infarction, stroke, and acute kidney injury.

Despite the important discoveries reported in these two outstanding pieces, several open questions remain to be answered to advance ferroptosis research in the clinic. First and most importantly, ferroptosis encompasses a too-broad conglomerate of individual cell death modalities. Not all “ferroptotic” cell death is controlled by GPX4 or FSP1. Second, the use of tool compounds such as RSL3 may not at all be specific for GPX4, but target other selenoproteins. Third, iron uptake may represent a common hallmark of all conditions referred to as ferroptosis, but transferrin may not sufficiently explain the import of iron to specific intracellular compartments. Fourth, it is entirely unclear why iron chelators would fail in clinical trials in which “ferroptosis” was claimed to represent the causative driver. Fifth, how would a clinical trial to inhibit ferroptosis be designed without an appropriate biomarker, and finally, how could venture capitalists be convinced to provide funding for such endeavors? For the latter, they shall once again ponder on the clinical relevance of “ferroptosis”.
